# A model for the use of blended learning in large group teaching sessions

**DOI:** 10.1186/s12909-017-1057-2

**Published:** 2017-11-09

**Authors:** Cristan Herbert, Gary M. Velan, Wendy M. Pryor, Rakesh K. Kumar

**Affiliations:** Department of Pathology, School of Medical Sciences, Faculty of Medicine, UNSW Sydney, Sydney, 2052 Australia

**Keywords:** Blended learning, Online learning, Large-group teaching, Higher education, Flipped classroom

## Abstract

**Background:**

Although blended learning has the potential to enhance the student experience, both in terms of engagement and flexibility, it can be difficult to effectively restructure existing courses. To achieve these goals for an introductory Pathology course, offered to more than 250 undergraduate students at UNSW Sydney, we devised a novel approach.

**Methods:**

For each topic presented over 2–3 weeks, a single face-to-face overview lecture was retained. The remaining content that had previously been delivered as conventional lectures was converted into short (12–18 min) online modules. These were based on lecture slides with added animations/highlights, plus narration using edited excerpts of previous lecture recordings. The modules also incorporated interactive questions and review quizzes with feedback which used various question types. Modules were developed in PowerPoint and iSpring and uploaded to Moodle as SCORM packages. Each topic concluded with an interactive large-group session focussing on integration of the content, with in-class questions to which students could respond via the Echo360 Active Learning Platform (ALP). Overall, more than 50% of face-to-face lecture time was replaced by online modules and interactive large-group sessions. Quantitative evaluation data included usage statistics from 264 students and feedback via online survey responses from 41 students. Qualitative evaluation data consisted of reflective commentaries from 160 student ePortfolios, which were analysed to identify factors affecting learning benefits and user acceptability.

**Results:**

All of the modules were completed by 74% of students and on average, 83.1% of students eventually passed the optional review quizzes. Notably, 88.4% of students responded to in-class questions during the integration and feedback sessions via the ALP. Student reflections emphasised that the modules promoted understanding, which was reinforced through active learning. The modules were described as enjoyable, motivating and were appreciated for their flexibility, which enabled students to work at their own pace.

**Conclusions:**

In transforming this introductory Pathology course, we have demonstrated a model for the use of blended learning in large group teaching sessions, which achieved high levels of completion, satisfaction and value for learning.

**Electronic supplementary material:**

The online version of this article (10.1186/s12909-017-1057-2) contains supplementary material, which is available to authorized users.

## Background

Blended learning is defined as a combination of traditional face-to-face learning with online content or other activities supported by information and communication technology [1]. Different approaches to blended learning use a variety of techniques, including replacement or supplementation of lectures with recordings, the use of pre-class videos, online assessments, ePortfolios, wikis or online tutorials. Regardless of the techniques used, the rationale for blended learning is to engage students and enhance the learning process [2]. Currently, there is an increasing drive to incorporate blended learning into higher education worldwide [3].

With traditional face-to-face teaching, much in-class time is dedicated to information transfer from a content expert to a novice student. The extent to which students engage with the content is highly variable, although small-group teaching certainly provides opportunities for direct interaction between the learner and the teacher, potentially enabling the student to obtain immediate feedback and clarification. However, large-group sessions such as lectures are often relatively impersonal [4] and students may find this approach is not ideal for their learning [5, 6]. Possible advantages of fully online courses are that they can be student-focussed, more flexible and may promote self-directed learning. However, fully online learning may have limited opportunities for student-teacher interaction and students may feel isolated, which may contribute to reported lower ratings for such courses [7]. Nevertheless, a meta-analysis of online learning in the health professions found little difference between the effectiveness of online and face-to-face formats [8]. Regardless, the relative effectiveness of online and traditional learning approaches is an area of ongoing controversy [7, 9, 10].

Well-designed blended learning has the potential to achieve the best of both face-to-face and online learning [11]. Blended learning maintains student-teacher interaction and peer learning, but can be more flexible by providing some content online and reducing the number of hours that students are required to be in class [12]. In addition, blended courses aim to enhance student interaction and engagement by providing content in a variety of different formats [13]. Millennials in particular may prefer blended learning, because they may be better versed in technology compared to previous generations of students [14].

It is important to recognise that simply supplementing face-to-face with online activities may be insufficient [15]. However, providing some of the content online may free time during classroom sessions for meaningful face-to-face interactions and other potentially engaging activities that reinforce and extend learning in the online environment [16].

Implementing blended learning well has several challenges [17]. Firstly, despite the many potential advantages, the success of blended learning approaches is dependent upon motivated teachers and students [17]. Academic staff may be hesitant to adopt blended learning due to the lack of technical skills and the increased workload required to develop resources for blended curricula. Early adopters may also have difficulty in finding the necessary institutional support [5]. Students need to develop appropriate discipline and time-management skills [18], and some may find it difficult to adapt to the new teaching format [19]. Other potential issues relate to infrastructure and support. Institutions need to ensure that the required hardware and internet capability are in place [19] and that there is adequate information technology support, because providing content online introduces the potential for technical issues to cause major teaching disruptions. There are also specific challenges associated with incorporating blended learning into existing courses. These include converting content into a suitable online format and determining how best to utilise in-class time. Difficulties may also arise if institutions lack specific policy on how blended courses should be run [13]. These challenges are compounded for large courses or courses with high information content [20].

A number of approaches have been reported which may help to guide academics who are designing new blended courses or blending existing courses [20–25]. There is considerable variation in both style and complexity, as well as variation in how well these approaches have been received by students [26, 27].

We developed an approach to radically transform an introductory Pathology course for more than 250 undergraduate students at UNSW Sydney. The previous version of this course included some online activities, such as adaptive tutorials and quizzes. However, the majority of the course content was delivered using traditional face-to-face lectures. We replaced over 50% of these lectures with online modules created using PowerPoint (Microsoft, Seattle WA) and iSpring Pro (iSpring Solutions, Alexandria VA). Modules incorporated narration in the form of edited excerpts of previous lecture recordings, slides that included animations/highlights, together with trigger questions and quizzes using several questions types. The remaining in-class time was dedicated to overview lectures and face-to-face technology-enhanced formative assessment sessions [28] that provided progressive feedback and were more engaging. The purpose of this study was to evaluate our approach to blended learning in terms of student utilisation of online modules and interactive large-group teaching sessions as well as student perception of the role of online resources for their learning.

## Methods

The project was reviewed and approved by the UNSW Human Research Ethics Committee (HC15134/HC16636). All students were provided with Participation Information Statements outlining that participation in the study was voluntary, that any feedback provided would not include identifying information and that their decision to participate (or not) would have no impact on their course outcome. Informed written consent was obtained from participants prior to the collection of feedback and survey data. Online modules were made available to all students regardless of whether they consented to participate in the study.

### Course structure

**Fig. 1:**
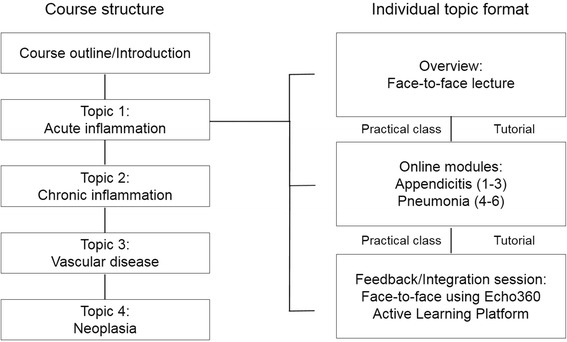
Blended Learning in the introductory Pathology course. Chart illustrating the overall course structure and the blended learning format used for each topic (using Acute Inflammation as an example topic). Overview lectures, online modules and Integration/Feedback sessions were taught over 2–3 weeks along with face-to-face tutorial and practical classes

The introductory Pathology course was delivered over 12 weeks to more than 250 students in their second year of Medical Science and Exercise Physiology Programs at UNSW Sydney. For the first 2 weeks, we retained the traditional lecture format, to acquaint students with the course structure, assessment details and introductory content. Thereafter, a consistent blended approach was applied to each of the 4 major topics (acute inflammation, chronic inflammation, vascular disease and neoplasia) addressed during the course (Fig. 1). Overall, we replaced more than 50% of the conventional face-to-face lectures with online modules and interactive large group sessions. Importantly however, no changes were made to the existing highly interactive weekly small-group tutorials and practical classes.

### Topic format

For each topic, learned over 2–3 weeks, a single face-to-face overview lecture was retained.

This lecture provided a broad overview of the topic, with key information and learning objectives. The lecture was recorded using the UNSW Echo360 (Reston, VA) system and made available via Moodle, the UNSW learning management system (LMS). The remaining content was provided as sets of short (12–18 min) online modules. Modules were based on PowerPoint presentations previously used for full-length lectures, with narration in the form of edited excerpts of previous lecture recordings. The free software Audacity (Audacity Inc., Renton WA, version 2.0.6) was used to edit and enhance audio clips. The slides were enhanced with animations and highlights which were synchronised with the audio within iSpring Pro (version 8.3.1). In addition, the modules included links to additional online resources, interactive questions and review quizzes using multiple question types with feedback. Typically, each 50-min lecture was converted into a set of 3 modules which focussed on a pathological process (e.g. the immune response) or an example of a specific disease (e.g. acute appendicitis).

HTML5 modules, able to be viewed in a browser on any device, were generated from PowerPoint presentations using iSpring Pro and were uploaded to Moodle as SCORM packages. After the relevant overview lecture, students were given unrestricted access to all of the modules associated with the topic.

Importantly, the review quizzes within the modules provided students with immediate feedback after each attempt. Completion of the quizzes within each module was not compulsory, and there was no limit to the number of attempts permitted for each quiz, as the scores obtained did not count towards the overall course mark. However, the module was not marked complete within the LMS until students obtained a score of 80% or more. The last screen in each set of modules included a link to a Perceived Utility of Learning Technologies Scale (PULTS) survey which had previously been developed and validated within the UNSW Faculty of Medicine’s Blended Learning Project (Velan et al., unpublished). The PULTS survey assesses factors that influence perceptions of and engagement with online learning resources, and is useful for evaluating online learning technologies (Additional file 1). Surveys were made available online via the Qualtrics survey engine (Qualtrics, Provo UT) and included Likert scales to assess features of the modules including whether they improved understanding of the topic, enhanced motivation and met student needs for flexibility in learning. The surveys also asked students to rate their understanding of the topic both before and after using the relevant modules, what students liked most about the specific modules, and what they would like to see changed.

Each topic concluded with an interactive large group formative assessment session which focussed on integration of the topic content. These “integration and feedback sessions” used the Echo360 Active Learning Platform (ALP) to present in-class questions, including questions related to relevant case studies, to which students could respond anonymously using their laptops, tablets or mobile phones. Immediate feedback was then provided by the lecturer, which was tailored to the overall class pattern of student responses.

Student scores from the integration/feedback sessions also did not contribute to the overall course mark. However, to encourage engagement with these sessions, 5% of the overall course mark was awarded to students who responded to at least two-thirds of the questions in those sessions. To further promote engagement, these sessions were not recorded (unlike the overview lectures), thus the benefits could only be obtained by attendance in person.

### Quantitative data

Student usage of the online modules was determined by data obtained from the SCORM packages and was reported as total attempts, completion rates for each module as well as the average number of attempts per module for each student. Student satisfaction with the online modules was initially assessed using a PULTS survey at the end of each set of modules.

### Qualitative data and analysis

Textual data was derived from commentaries recorded in student online ePortfolios [29], in which they were asked to ‘reflect critically on the use of online modules for your learning in this course’. Thematic analysis was performed on ePortfolio responses received from 160 students.

Aided by the use of NVivo software (QSR International, London UK), we used a descriptive phenomenological approach with coding of phenomena to hierarchical nodes, followed by data reduction to identify emergent themes.

### Statistical analysis

Data are presented as mean ± SEM or median ± interquartile range as appropriate. Student’s *t*-tests were employed to assess differences in usage data and course outcomes, while Mann-Whitney tests were used to assess differences in categorical data from the survey. A *P* value of < 0.05 was considered significant.

## Results

Overall, 29 modules and 4 integration/feedback sessions were created, which were delivered to 264 Pathology students in Semester 2, 2016. Throughout the semester, no significant technical issues were encountered. The time required for students to complete all lectures, online modules and integration/feedback sessions in the semester was 23.25 h (lectures + integration/feedback sessions + 29 × 15-min modules).

### Online modules

By the completion of the course, 74% of enrolled students (*n* = 264) had attempted all 29 online modules and 85% of enrolled students had completed 90% or more of the modules.

**Fig. 2:**
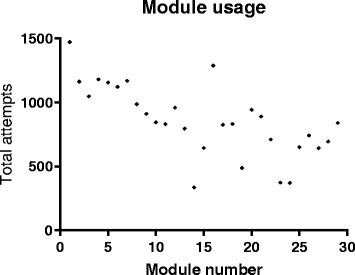
Overall usage of each of the online modules. Graph showing the total number of times each module was attempted throughout the semester. Modules are numbered in the order of release

For each module, the total number of attempts was considered to be a measure of usage [30]. Initial usage was very high, with the first module attempted a total of 1472 times (5.6 attempts per student) and over 1000 attempts for each of modules 2–6 (Fig. 2). Usage of modules released in the 2nd half of the semester was noticeably lower with an average of 704.3 ± 61.2 (mean ± SEM) attempts for each of the final 16 modules. This was in part related to comparatively few attempts of four specific modules (14, 19, 23 and 24), for which there was an average of only 391.5 ± 33.3 attempts.

**Fig. 3:**
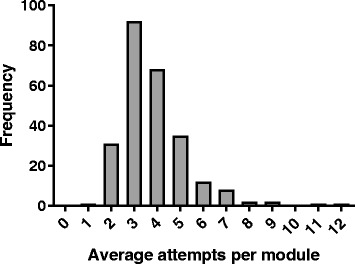
Usage of online modules by individual students. Histogram showing the average number of times modules were attempted by each student

There was considerable variation in the interaction with the online modules by individual students (Fig. 3). While one student attempted each module an average of only 0.10 times (3 attempts in total over the semester), another attempted each module an average of 11.7 times (340 total attempts). Overall, students attempted each module an average of 3.3 ± 0.1 times. Students who completed at least 90% of the modules also made significantly more attempts than students who completed fewer than 90% of the modules (101.3 ± 2.8 total attempts compared to 55.9 ± 5.5 total attempts; *P* < 0.001).

**Fig. 4:**
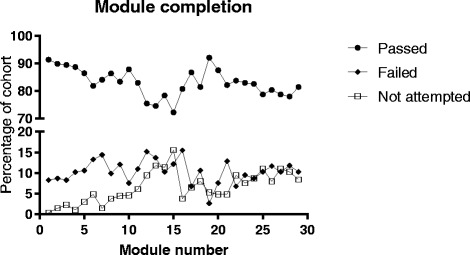
Final completion rates for all online modules. Graph showing the percentage of enrolled students (*n* = 264) who by the end of the course had accessed the module and passed the review quiz, accessed the module but not passed the review quiz or had not accessed the modules

Based on the SCORM data for each of the modules, the majority of students repeated the modules until they were able to achieve a pass score of at least 80% in the final review quizzes (Fig. 4). The modules were eventually completed (i.e. review quiz was passed) by 83.1% of students, but an average of 6.5% of students did not access the modules.

### Integration and feedback *sessions*

Student attendance at each of the face-to-face integration/feedback sessions was consistently high. Average attendance for the 4 large-group sessions was 88.4 ± 1.6% of enrolled students (*n* = 264). In contrast, only 54.2% of the cohort attended an end-of-semester revision session for which engagement was not linked with the course marks (not shown).

### Student feedback

**Fig. 5:**
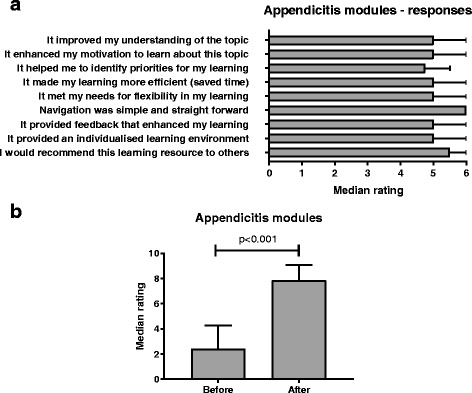
Student engagement and satisfaction as indicated by the PULTS survey. Overall rating (median plus interquartile range; *n* = 41) of student perception of the online modules used in the introductory course. Students indicated the extent to which they agreed with each of the statements by clicking on stars, with 0 indicating no agreement, and 6 indicating strong agreement (**a**). Median and interquartile range (*n* = 41) of self-reported “understanding of the topic” before and after using the online modules. *P* < 0.0001 by two-tailed Mann-Whitney test (**b**)

The voluntary PULTS survey was completed by 41 students at the end of the first set of modules. Survey data indicated high overall student satisfaction with the online modules. Importantly, students agreed strongly that the modules provided “feedback that enhanced learning” and “an individualised learning environment” (Fig. 5a). There was a significant increase in student’s self-reported rating of their understanding of topics after using the online modules (*p* < 0.001; Fig. 5b).

### Analysis of student reflections

Thematic analysis of 160 student ePorfolio reflections identified several positive attributes of the online modules (Table 1). The capacity of the modules to build understanding was strongly borne out in the reflective comments. Furthermore, some students stated that the contextual questions and quizzes required them to exercise critical thinking and in some cases promoted meta-learning i.e. the transferable skills associated with students’ understanding about their own learning processes. Motivation to learn was regularly articulated in terms of enjoyment and satisfaction that students experienced as they successfully answered questions and completed quizzes, thereby affirming their learning. Students commented that the modules enabled them to identify priorities in their overall study plan, and helped them to identify the areas where they most needed to focus their efforts. Time-efficiency was a prominent theme, primarily attributed to the organisation and succinct nature of the material presented. The modules offered flexibility by facilitating the ability to stop, and replay as needed in order to digest the material, and also because students were able complete them at convenient times and places. Students were strongly positive regarding navigation and organisation of the material. Feedback, which was continuously provided throughout the modules, emerged as the most popular and valuable features of the modules, being useful for self-evaluation and checking understanding. Finally, students commented that individualisation was achieved through the use of multiple modalities to cater for a variety of learning approaches.

**Table 1:** Positive aspects of the online modules identified from thematic analysis of student ePortfolio reflections

Identified theme	Representative student reflection
Promoting understanding	*“In addition to the normal lectures provided, online modules bring together and make sense of newly taught information”*
Promoting deeper learning	*“Having online modules as opposed to traditional lectures forces students to embrace independent learning and allows us to develop the skills of ‘how to learn’.”*
Motivation	*“I was motivated to complete these modules because it was quick to go through and I thoroughly enjoyed the audio recording alongside with slides. The imagery was very intriguing and this was reflected in my motivation to perform well.”*
Identifying priorities	*“With the amount of information covered in lectures, I sometimes struggle identifying whether certain parts are important or not… However, the online modules allow me to prioritise which parts of the topic are required whilst providing a good amount of detail.”*
Efficiency	*“The information in the slides was concise and very well structured making the process of studying the course very efficient.”*
Flexibility	*“…to my rescue come the online modules, which can be done when and if I am ready to learn, usually on a Sunday afternoon in the sun.”*
Navigation	*“I enjoy that I’m able to pause throughout the modules because it gives me plenty of time to understand and take notes, rather than in a lecture where I feel I miss things because I sometimes struggle to keep up with note taking.”*
Feedback	*“The quizzes can be hard, but they provide really good feedback on how I’m actually going and whether I’ve critically understood what was presented to me.”*
Individualisation	*“I think having a variety of mediums where the same information is presented to us, whether it be the tutorials, macropathology labs or online modules, has given me choices to suit to my study habits and study needs, but also helped me experiment with the learning style I am most accustomed to.”*

Thematic analysis also identified several potential problems with the modules (Table 2). Convenience and flexibility came at a cost for some students who tended to procrastinate. Competing demands of other study and personal commitments presented real challenges for some students in terms of time management. Lack of personal interaction with individuals was often seen as one of the negative aspects of learning using the modules, due to the absence of much-valued social engagement and lack of opportunity to ask questions. However, some found that they could make a note of their questions and subsequently raise them in live tutorials. Also, a significant criticism of the modules was that they were not always well-aligned with live lectures and tutorials in terms of content and timing. Some felt that the sequence and timing of release was confusing and this detracted from their ability to integrate material across the platforms.

**Table 2:** Negative aspects of the online modules identified from thematic analysis of student ePortfolio reflections

Identified theme	Representative student reflection
Alignment with other activities	*“One aspect I am finding disjointed is the flow between module release, overview, integration lectures, practicals and tutorials. By the time the [integration/feedback session] comes round, it feels like a long time since those modules where done”*
Lack of detail	*“I felt that not all of the modules provided enough information. That is, in order to gain a deeper understanding, I needed to use other resources such as the textbook and various websites”*
Flexibility	*“The only down side to the online modules I found was that the freedom to complete the modules meant that I often left them to the last minute before completing them, to my own detriment.”*
Learning environment	*“I find the university environment to be quite motivating and harmonious with students and the positive learning atmosphere. Yet, with online modules, I find this to be missing.”*
Personal interaction	*“…the downside I have found with these modules is that because it is all online, there is no way to interact with the lecturer and ask them questions if I get stuck on a certain piece of content”*
Accessibility	*“…online modules do require good internet connections, otherwise there are always pop up signs telling you that your internet connection is unstable. This is somewhat disruptive to our learning.”*

Other less frequently cited concerns included lack of detail in the modules, a need for more text to accompany the histological images on slides and difficulty streaming online content from off-campus internet connections.

### Course outcomes

At the completion of the course, students’ overall performance appeared to be associated with their level of online module usage throughout the semester. The average course mark achieved by students who completed at least 90% of the modules was significantly higher than by students who completed less than 90% of the modules (72.4% compared to 56.5%; *p* < 0.0001). Notably, 21.1% of the students who completed less than 90% of the modules failed the course, compared to only 4.5% of students who completed at least 90% of the modules.

## Discussion

In this study, we have described an approach for incorporating blended learning into a large introductory Pathology course. For each of the 4 topics in the course, content was provided using a single face-to-face overview lecture, followed by 6–8 interactive online modules. Each topic concluded with a face-to-face integration and feedback session, which included some relevant case studies and in-class questions delivered using the Echo360 Active Learning Platform. The course achieved more effective delivery, was student-focused and was highly interactive, but was not designed to provide individualised instruction [31]. In the new format, the minimum time required to complete all online modules and face-to-face activities was 23.25 h, compared to 26 h in the traditional lecture format.

Development of this combination of face-to-face and online sessions enabled us to successfully incorporate blended learning into an existing course, while taking full advantage of previous resources and maintaining the strengths of the course in its previous form. The combination approach is similar to one that was used for a newly-developed course at the University of Otago, which was well received by medical students [27]. We believe this is an effective model for the introduction of blended learning for both new and existing large group university courses. Student reflections provided evidence that the various course components were used synergistically in our model, offering a holistic learning experience.

Our approach to blended learning shares similarities with the inverted classroom model (ICM) as both approaches include a classroom-learning phase which assimilates integrates and builds on knowledge obtained during a self-directed phase [24, 25]. However, in our approach, we have retained an in-class overview lecture to help students understand key concepts prior to the online modules.

Often in blended courses, recorded lectures are used to provide course content [32]. While this approach can be useful, there may be limitations particularly for engineering, science and medicine courses which involve highly technical content or demonstrations [32]. For the Pathology course, we aimed to develop highly engaging modules by incorporating excerpts from lecture recordings along with PowerPoint slides containing animations and highlights, which were synchronised with the audio. Modules also contained supplementary notes and links to additional sources of information such as videos and textbooks. Students often commented that the use of multiple modalities was engaging and catered for a variety of learning approaches.

Importantly, 25 of the 29 modules also contained review quizzes, with a variety of text and image-based questions. Although a range of different software is available for creating online content, we chose iSpring Pro because this has full PowerPoint integration, excellent user control of playback and easy intercalation of quizzes. We estimate that each module took approximately 20 h to develop and test. Modules were published as SCORM packages, which enabled the collection of usage and completion data via the learning management system. The quizzes were the most regularly cited elements of the modules that led to deeper understanding of the material and identification of priorities for revision. The interactivity stimulated active learning processes which aided concentration and retention of knowledge.

Online modules released at the beginning of the semester were used extensively, with over 1000 individual attempts for each of the first 7 modules. Interaction with subsequent modules decreased markedly, possibly because the novelty of the new approach had faded. Use of the modules released in the second half of the semester was more consistent except for four modules for which the number of attempts was unusually low. It was notable that these were the only 4 modules that did not contain a review quiz, suggesting that the quizzes were the reason students were making multiple attempts at the other modules. Another possible explanation for non-completion was that there was some confusion about the sequence of module delivery.

Quizzes were provided as a formative assessment to make the modules more engaging, but also with the aim of improving students’ satisfaction with the course and their performance in subsequent summative assessments [33]. Other studies have found that the ability of students to access the content at a time convenient to them may increase their satisfaction [34]. In our study, flexibility was one of the major factors that promoted student satisfaction, enabling students to complete the modules at their own pace at convenient times and locations. The counterpoint is that the flexibility provided by online delivery may increase the likelihood of some students falling behind [7]. A number of our students admitted that they tended to procrastinate in the face of other demands, though some found that the need to complete the modules actually helped them to reflect upon and address their time management.

Maintaining engaging face-to face sessions was a key component of our approach to blended learning for this course. We retained a single overview lecture at the beginning of each topic to provide students with key information. However, because didactic lectures may lead to suboptimal knowledge recall and poor development of critical thinking [22, 35], active learning techniques were also incorporated into many of these overview lectures. Each topic concluded with a large group face-to-face session which focussed on integration of the content and correction of any misconceptions. This was achieved using example case studies and a series of questions to which students could respond in-class using their mobile devices. We used relatively few case studies for our Science students studying Pathology, but recognise that if this approach was employed for medical students, it would be appropriate to further contextualise the questions with additional case studies.

In contrast to the overview lectures and online modules, which aimed to promote understanding of concepts, the large-group integration/feedback sessions required students to analyse new information and apply what they have learned previously to these new situations, and therefore aimed to achieve higher cognitive levels [36]. The question and answer technique used in the integration/feedback sessions aimed to enhance engagement and increase student satisfaction and participation in discussions [28, 37]. The combination of online modules and face-to-face sessions was found to be synergistic in terms of integration and reinforcement of knowledge. On average, over 88% of enrolled students (*n* = 264) attended the integration/feedback sessions, an outstanding percentage at a time when student attendance at lectures is declining steadily and is often below 50%. This may be a result of the engaging format of the ALP sessions [20] or the fact that students were awarded a small component of the course mark (5%) for providing responses to the questions in these sessions. At the end of the semester, an interactive review session which was not linked to the final course mark was attended by only 54.2% of students (data not shown) suggesting that having marks associated with the activity strongly influenced students’ decisions to attend. The integration/feedback sessions were intended as formative assessments, which is why marks were not deducted for incorrect responses to the in-class questions. In future, it may be possible to increase engagement further by linking student responses during these sessions to course outcomes.

A PULTS survey was used to initially evaluate student perceptions of how the online modules contribute to their learning. This survey was previously developed and validated within a Faculty-wide Blended Learning Project. Importantly, students agreed that the modules contributed to their learning, provided feedback which enhanced learning and provided an individualised learning environment. However, we recognise that the data are incomplete, because the links to the surveys were provided at the end of the modules, so that students who did not attempt the modules would not have completed the survey. Also, while 235 students provided consent, only 41 survey responses were received after the first topic. As a result, data from the PULTS survey are likely to represent the opinion of only the most motivated students. Fortunately, the reflective comments in ePortfolios provided a rich source of student feedback data. Online ePortfolios were introduced into the course in 2012 in order to promote students’ development of reflective practice and to document their development of professional skills [29]. Students described the modules enjoyable and motivating and appreciated their flexibility, which enabled students to work at their own pace, at convenient times and places.

Other studies have found that a blended approach to learning is not appreciated by all students [38], mainly because of reduced student/teacher interaction and online connection issues. In our study, some students commented in their ePortfolio reflections that fear of losing opportunities for student/teacher interaction and frustration due to not being able to ask questions while completing modules were significant concerns, though this was often overcome after commencing the program. This highlights the importance of maintaining opportunities for students to ask questions during face-to-face sessions.

A minority of students encountered internet connectivity issues while accessing the online content from home. It is likely that will be less frequent in the future as more people acquire high-speed internet connections at home [39], provided that the infrastructure can keep up with the increasing demand [40].

Students clearly appreciated the review quizzes in the modules and many suggested that there should be more questions. This was reinforced by the low level of usage of the 4 modules which did not contain a quiz.

It was interesting to note that students who attempted at least 90% of the modules achieved a significantly higher final mark than students who did not. It is possible that engagement with the modules contributed to the higher marks, but alternatively it may simply be that high-achieving students engage more thoroughly with course material.

A limitation of our study is that we did not compare student performance before and after introduction of blended learning. Numerous confounding factors make it difficult to perform robust studies of this type. However, published reports suggest that there is little if any significant difference in the performance of students undertaking face-to-face or hybrid courses [36, 41].

## Conclusions

In the current study, we successfully transformed an introductory Pathology course by replacing over 50% of the face-to-face lectures with interactive online modules, to be able to use the remaining in-class time for more meaningful and engaging activities. In fact, the modules proved to be meaningful and engaging in themselves, and worked synergistically to provide a holistic experience that was highly effective in enabling and reinforcing student learning. We observed a very high level of usage of the online modules released early in the semester and lower rates of usage with later modules, possibly due to student time-management issues, and some confusion about sequencing of module delivery in relation to other components.

Engagement during face-to-face integration/feedback sessions was high. While some students were concerned that the “lecturer-student interaction” may be impaired, most found that the blended approach maintained engagement primarily due to the use of “interactive quizzes” and the flexibility afforded by being able to work at their own pace at convenient times and locations. Our approach was demonstrated be an effective model for introducing blended learning in large group teaching sessions into existing courses.

## Additional file

Additional file 1: Perceived Utility of Learning Technologies Scale (PULTS) survey. An example of the questions from the online Perceived Utility of Learning Technologies Scale (PULTS) survey (PDF file). Links to online surveys, via the Qualtrics survey engine, were presented to students at the completion of online modules relevant to specific topics (e.g. Acute Inflammation). (PDF 234 kb)

## Abbreviations

ALP: Active learning platform
LMS: Learning management system
PULTS: Perceived utility of learning technologies scale
SCORM: Shareable content object reference model
