# Supplementary material for: A model for the use of blended learning in large group teaching sessions
Source: BMC Med Educ. 2017 Nov 9;17:197. doi: 10.1186/s12909-017-1057-2 (PMC5680783; doi:10.1186/s12909-017-1057-2)
Supplement: Additional file 1: — Perceived Utility of Learning Technologies Scale (PULTS) survey. An example of the questions from the online Perceived Utility of Learning Technologies Scale (PULTS) survey (PDF file). Links to online surveys, via the Qualtrics survey engine, were presented to students at the completion of online modules relevant to specific topics (e.g. Acute Inflammation). (PDF 234 kb) [file 12909_2017_1057_MOESM1_ESM.pdf]

Perceived Utility of Learning Technologies Scale (PULTS) survey

Please indicate to what extent you agree with each of the statements below regarding this Acute Appendicitis Module Set:

It improved my understanding of the topic	☆☆☆☆☆☆
It enhanced my motivation to learn about this topic	☆☆☆☆☆☆
It helped me to identify priorities for my learning	☆☆☆☆☆☆
It made my learning more efficient (saved time)	☆☆☆☆☆☆
It met my needs for flexibility in my learning	☆☆☆☆☆☆
Navigation was simple and straight forward	☆☆☆☆☆☆
It provided feedback that enhanced my learning	☆☆☆☆☆☆
It provided an individualised learning environment	☆☆☆☆☆☆
I would recommend this learning resource to others	☆☆☆☆☆☆

Gender:

Male ☐

Female ☐

Please rate your understanding of the topic:

	0	1	2	3	4	5	6	7	8	9	10
BEFORE you used this Acute Appendicitis Module Set											
AFTER you used this Acute Appendicitis Module Set											

Please comment on what you liked most about this Acute Appendicitis Module Set:

Please comment on what you would like to see changed in this Acute Appendicitis Module Set:

Professor Gary M. Velan

UNSW Sydney
